# A proof-of-concept study of vicarious extinction learning and autonomic synchrony in parent–child dyads and posttraumatic stress disorder

**DOI:** 10.1038/s41598-023-41722-0

**Published:** 2023-09-11

**Authors:** Sara A. Heyn, Grace George, Emily Hamm, Christy Olson, Julia H. Harari, Marie-France Marin, Mohammed R. Milad, Ryan J. Herringa

**Affiliations:** 1 Department of Psychiatry, University of Wisconsin-Madison School of Medicine and Public Health, 6001 Research Park Blvd., Madison, WI 53719 USA; 2 McLean Hospital, Harvard Medical School, Belmont, MA USA; 3 Department of Counseling Psychology, University of Wisconsin-Madison, Madison, WI USA; 4 School of Medicine and Public Health, University of Wisconsin-Madison School of Medicine and Public Health, Madison, WI USA; 5 Department of Psychology, Université du Québec à Montréal, Montreal, QC Canada; 6 Department of Psychiatry and Behavioral Health, McGovern Medical School, University of Texas Health at Houston, Houston, TX USA

**Keywords:** Post-traumatic stress disorder, Extinction, Fear conditioning

## Abstract

Though threat-extinction models continue to inform scientific study of traumatic stress, knowledge of learning and extinction as mechanisms linking exposure to psychopathology remains critically limited among youth. This proof-of-concept study advances the study of threat-extinction in youth by determining feasibility of electrodermal stimulation (EDS), vicarious extinction learning via their parent, and social threat learning in pediatric PTSD (pPTSD). Typically developing (TD) and PTSD-diagnosed youth in 45 mother–child dyads completed an extinction learning paradigm. The use of EDS was first investigated in a cohort of TD youth (n = 20) using a 2-day paradigm without vicarious extinction, while direct (for TD and pPTSD) and vicarious (for pPTSD) extinction were investigated in a 3-day paradigm (n = 25). Threat acquisition and extinction were monitored using skin-conductance response (SCR) and behavioral expectations of EDS. Using Bayesian modeling to accommodate this pilot sample, our results demonstrate: (1) EDS-conditioning to be highly feasible and well-tolerated across TD and trauma-exposed youth, (2) Successful direct and vicarious extinction learning in trauma-exposed youth, and (3) PTSD-associated patterns in extinction learning and physiological synchrony between parent–child dyads. In summary, these novel approaches have the potential to advance translational studies in the mechanistic understanding of parent–child transmission of risk and youth psychopathology.

## Introduction

Threat extinction learning has been used in animal models and humans to study mechanisms of the development and flexibility of memory since the 1920’s^1,2^. The study of vicarious or observational learning, the ability to learn through others’ experiences, has further been well documented across species^3^. This ability to acquire a threat association without direct experience of aversive stimuli is an adaptive mechanism that contributes to survival, and research on social learning suggests that youth (ages 6–10 years-old) may primarily learn threat and safety associations through parental observation^4^. Disruption of this learning process may contribute to child psychopathology^5^. For example, parent anxiety has been found to predict increased threat learning and hyperactive neural responses to threat in children^6^. While genetic factors likely contribute to this association, parental modeling of threat and safety discrimination is also thought to play a role^7,8^. Thus, understanding the contribution of parental and adult modeling in safety learning, and how differences contribute to anxiety and threat disorders in youth is of great importance. If differences in vicarious learning are apparent in vulnerable populations, like those exposed to maltreatment, parsing out which processes are specifically affected (e.g., youth perception or interpretation of cues, increased threat learning, decreased threat extinction) could provide insight for novel and targeted treatments.

Threat learning paradigms have been salient targets when trying to characterize affective disorders like anxiety, depression, and PTSD^9^. Several specific processes involved in threat learning have been proposed as salient to PTSD and other threat disorders, including enhanced acquisition, stimulus (over-)generalization and impaired extinction learning and extinction memory^2,10,11^. While these processes are well-documented in adult humans^9^, little work has been done so far investigating mechanisms of threat and extinction learning in adolescent populations, let alone in youth that have been exposed to trauma and/or with affective disorders. To our knowledge, Marusak et al.^12^ and McLaughlin et al.^13^ are the only studies that have implemented an extinction learning paradigm in maltreated youth populations^12,13^. Both studies utilized short paradigms (either 1- or 2-days) and an aversive noise burst as the unconditioned stimulus (US). In maltreated youth, these studies reported blunted threat responses during acquisition^13^ and an overgeneralization of threat behaviors to all stimuli regardless of previous US pairing during extinction recall^12^. To our knowledge, however, no studies have examined the use of electrodermal stimulation (EDS; e.g. finger shocks) in youth as a US, which is commonly employed in both adult human and animal studies, nor the role of vicarious extinction learning in typically developing or psychopathology-affected youth.

One mechanism through which vicarious extinction may occur is through parent–child physiological synchrony. Synchrony is the temporally-matched coordination of responses between two people^14^. For parent–child dyads, synchrony is a critical method of learnt emotion regulation in children and a way to foster healthy attachments^15^. Physiological synchrony uses peripheral nervous system methods like skin conductance response (SCR) or heart rate variability (HRV) to evaluate the degree to which dyads are coupled^14^. While trauma may lead to differences in physiological, or autonomic, synchrony in youth and parents alike, how these variations affect real world behaviors like threat learning is still unknown^16,17^. Understanding the biological mechanism behind vicarious learning is crucial for understanding transmission of threat and safety cues between dyads, especially in those with threat-related disorders like PTSD.

The current study was adapted from a paradigm we recently implemented in typically developing (TD) children and their caregivers^18^. It is the first, to our knowledge, to test the feasibility and tolerability of using classic electrodermal stimulation (EDS) in youth, vicarious threat extinction in PTSD-affected youth, and potential mother–child dyadic physiological synchrony mechanisms underlying learning differences in youth with PTSD. Due to the a priori interest in the tolerability and feasibility of EDS in a developmental population, mother–child dyads across a large adolescent age range were recruited. We further explored alterations in threat learning and direct/vicarious extinction learning in pediatric PTSD to inform future hypothesis testing in larger samples of youth. Namely, we examined whether there are differences in extinction learning in youth with and without PTSD, as measured by SCR and self-reported US expectancy, and how parental threat extinction translates to youth extinction via observation and parent–child physiological synchrony.

Here, we tested the following a priori hypotheses: (1) All mother–child dyads, regardless of PTSD status in youth, will tolerate a threat paradigm using EDS equally or better than paradigms involving other aversive stimuli (e.g. air blast to the larynx, loud tones or white noise, or human screams), as evidenced by low study attrition; (2) Using EDS with parents and youth, we will be able to invoke anticipated physiological and behavioral indices of successful threat acquisition (i.e. increased arousal and expectancy of EDS) as well as both direct and vicarious threat extinction (i.e. decreased arousal and expectancy of EDS); and (3) We will detect PTSD-associated differences in arousal during vicarious extinction learning that may be mechanistically linked to aberrant physiological synchrony between parents and youth.

## Results

### Participant characteristics and EDS tolerability

**Figure 1:**
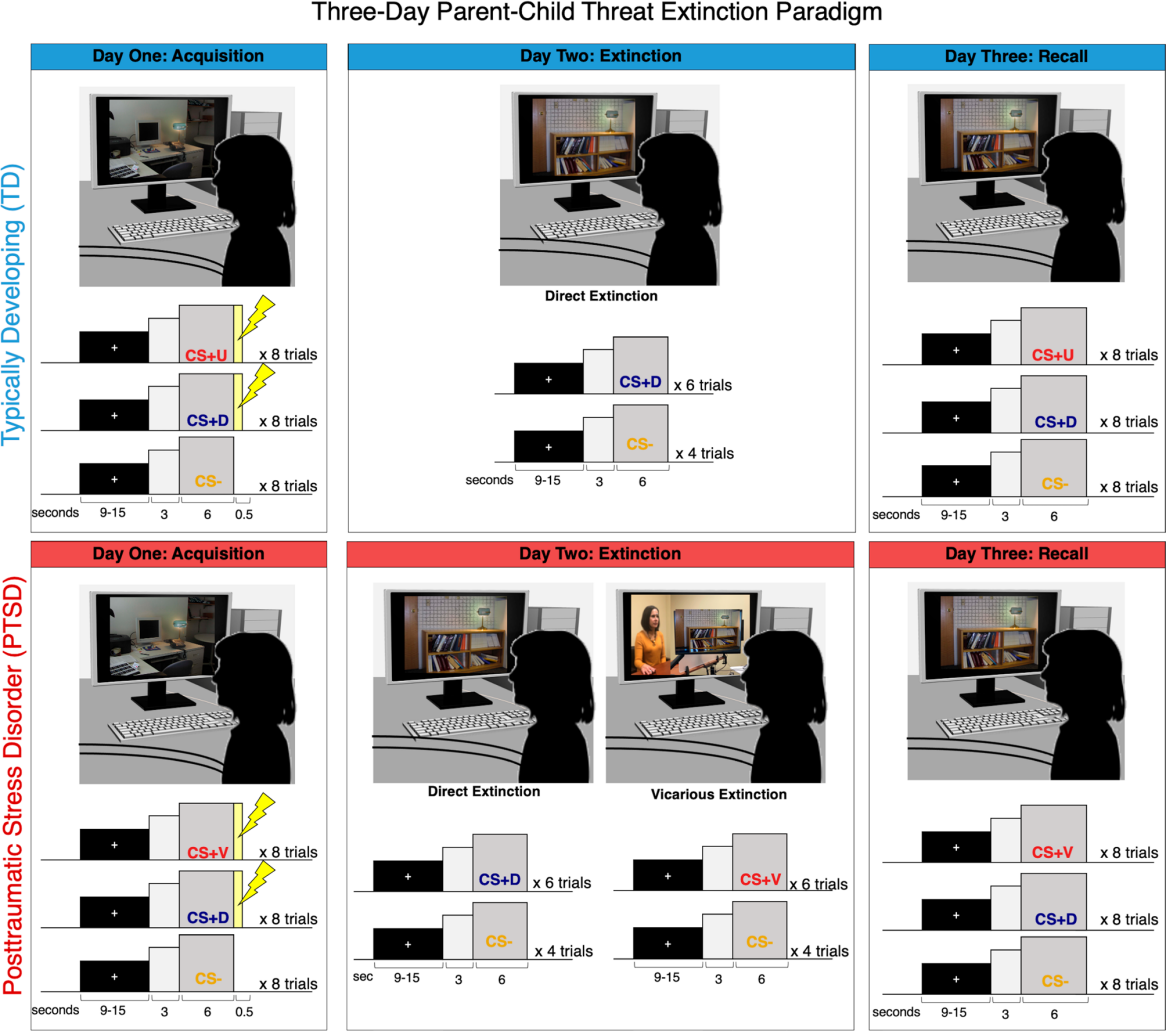
Observational threat extinction paradigm in parent–child dyads. Schematic of the phases of the full, three-day observational threat extinction paradigm. During each phase, CS presentation is preceded by a fixation cross for a jittered duration of 9–15 s, and a three second presentation of the scene without the CS light turned on (as pictured). During the second day, direct extinction is completed by all youth, while only PTSD youth completed direct and vicarious extinction with the order of presentation counterbalanced. Mothers will complete the same paradigm as youth, but will only ever complete direct extinction, leaving the unextinguished CS+ (CS + U). The US for all participants, electrodermal stimulation, is represented by a yellow lightning bolt. The number of trials for each stimulus and phase is noted, and presentation order was random across stimulus type in each phase. Informed consent was obtained to publish the identifiable image. *CS* conditioned stimulus, *CS−* unpaired CS, *CS + D* directly extinugished CS, *CS + V* vicariously extinugished CS, *CS + U* unextinguised CS.

**Figure 2:**
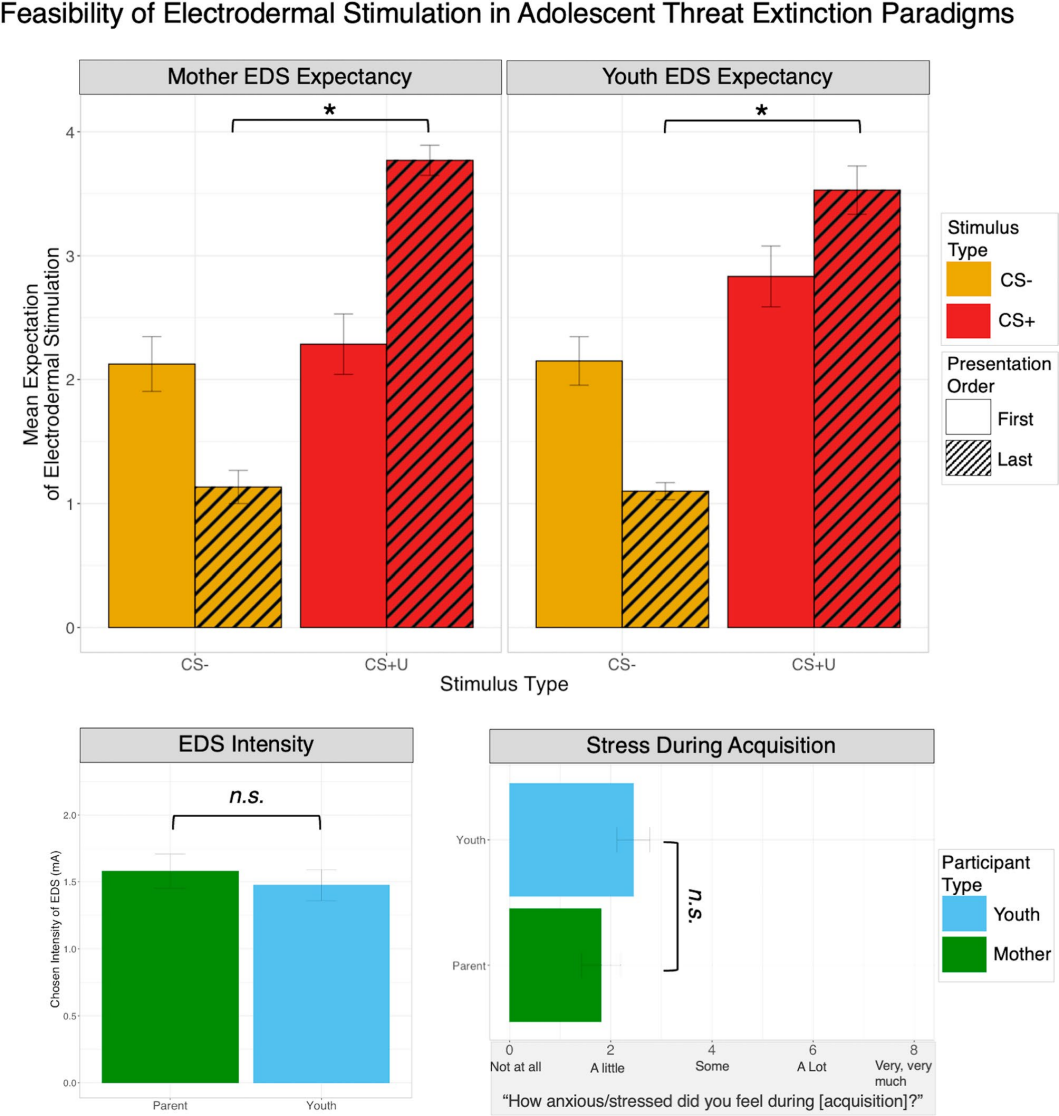
Feasibility of electrodermal stimulation (EDS) in adolescents and a three-day observational threat extinction paradigm in mother–child dyads. Within a smaller cohort of typically developing dyads (n = 20), all parent (mothers) and youth participants show increased behavioral expectancy of the EDS at the last presentation of the CS+ as compared to the CS−, and similar chosen EDS intensity and perceived stress during acquisition across parents and youth. *CS+* conditioned stimulus, *CS−* unpaired CS.

A total of 45 youth and their accompanying mother completed a threat conditioning paradigm, summarized in Fig. 1, split between an EDS tolerability cohort (n = 20) and a 3-day extinction learning paradigm, where TD youth (n = 10) completed direct extinction (with the other CS + unextinguished) and PTSD youth (n = 15) completed both direct and vicarious extinction. Demographics and clinical characteristics for each cohort are summarized in Table 1. First, 20 mother-youth dyads completed a preliminary 2-day threat extinction paradigm using EDS as the US. Here, youth and parent chose similar levels of EDS intensity (youth, *M* = 1.76 mA; parent, *M* = 1.73 mA; Youth > /Parent, *t*(31.9) = 0.08, *p* = 0.94) and low levels of self-reported stress during the acquisition phase using EDS (youth, *M* = 2.46; parent, *M* = 1.18; Youth > /Parent, *t*(31.4) = 1.26, *p* = 0.22). Using the EDS expectancy questionnaire, both youth and parents behaviorally exhibited successful threat acquisition (Fig. 2).

**Table 1:** Demographics and clinical characteristics.

	EDS tolerability	TD	PTSD
Demographics			
n	20	10	13
Child Age	14.12 (± 1.37)	10.81 (± 1.72)	13.73 (± 3.59)
Child Sex	9F	6 F	11 F
Child Pubertal Stage	–	2.28 (± 1.12)	3.35 (± 1.30)
Child EDS Intensity (mA)	1.76 (± 0.92)	2.16 (± 1.11)	1.97 (± 0.74)
Parent Age	46.71(± 4.49)	44.37 (± 6.89)	40.84 (± 7.89)
Parent EDS Intensity (mA)	1.73 (± 0.77)	1.96 (± 0.91)	2.22 (± 0.90)
Clinical characteristics			
Child PTSD Symptoms	–	–	50.08 (± 16.35)
Child Depression Symptoms	–	–	18.08 (± 8.89)
Child Anxiety Symptoms	–	–	31.67 (± 11.68)
Child Medication History (n)	–	–	9
Parent Psychiatric Diagnoses (n)	–	–	Current Internalizing (4) Past Internalizing (2) No past/current diagnosis (3) Unknown (4)

Parentheticals denote standard deviation. Child PTSD symptoms represent total UCLA PTSD Reaction Index (PTSD-RI) scores, child depression symptoms represent total scores of the Mood and Feelings Questionnaire (MFQ), and child anxiety symptoms represent total Screen for Child Anxiety-Related Emotional Disorders (SCARED) scores.

*TD* typically developing, *PTSD* posttraumatic stress disorder, *EDS* electrodermal stimulation.

During the threat acquisition phase, youth (Last CS+  > Last CS−, *t*(22.6) = 10.49, *p* < 0.001) and parents (Last CS+  > Last CS−, *t*(24.1) = 8.34, *p* < 0.001) reported higher EDS expectation at the last presentation of the CS+ as compared to the CS− (Fig. 2). Finally, among this cohort, we report 0% participant dropout. This cohort provided substantive evidence of feasibility and tolerability of using tactile electrodermal stimulation as a US in adolescent threat extinction paradigms.

Next, to isolate and individually characterize each phase of the paradigm while allowing for ample threat learning consolidation between phases, this paradigm was expanded to the full, 3-day threat extinction paradigm. Within this cohort, all youth participants had an average age of 12.46 (± 3.23) years, although PTSD youth were significantly older (PTSD > TD, *t*_(20)_ =  − 2.58, *p* = 0.02). Youth selected a stimulation level comparable to that of their parent (youth: *M* = 2.05 mA, *SD* = 0.90; parents: *M* = 2.11 mA, *SD* = 0.89; Youth > /Parent, *t*(_43)_ = − 0.21, p = 0.83). Youth’s stimulation level selection was not significantly correlated with age (*r*(21) = 0.26, *p* = 0.23). Furthermore, youth in the PTSD group did not differ in selected stimulation levels compared to TD youth (*t*_(32)_ = 0.45, *p* = 0.66). When combining all study cohorts, we report only a 4% dropout rate, where only 2 of 45 mother-youth dyads dropped mid-study. The two dropouts were both in the PTSD group due to self-reported boredom with the study (n = 1) and suspicion of ongoing abuse (n = 1). Notably, no participants in either group dropped out due to intolerance of the electrodermal US. Finally, in both TD and PTSD groups, performance during attentional control questions were high and did not significantly differ (TD: Parents, 95%, Youth, 89%; PTSD: Parents, 96%, Youth, 87%).

### Task validation in typically developing youth

**Figure 3:**
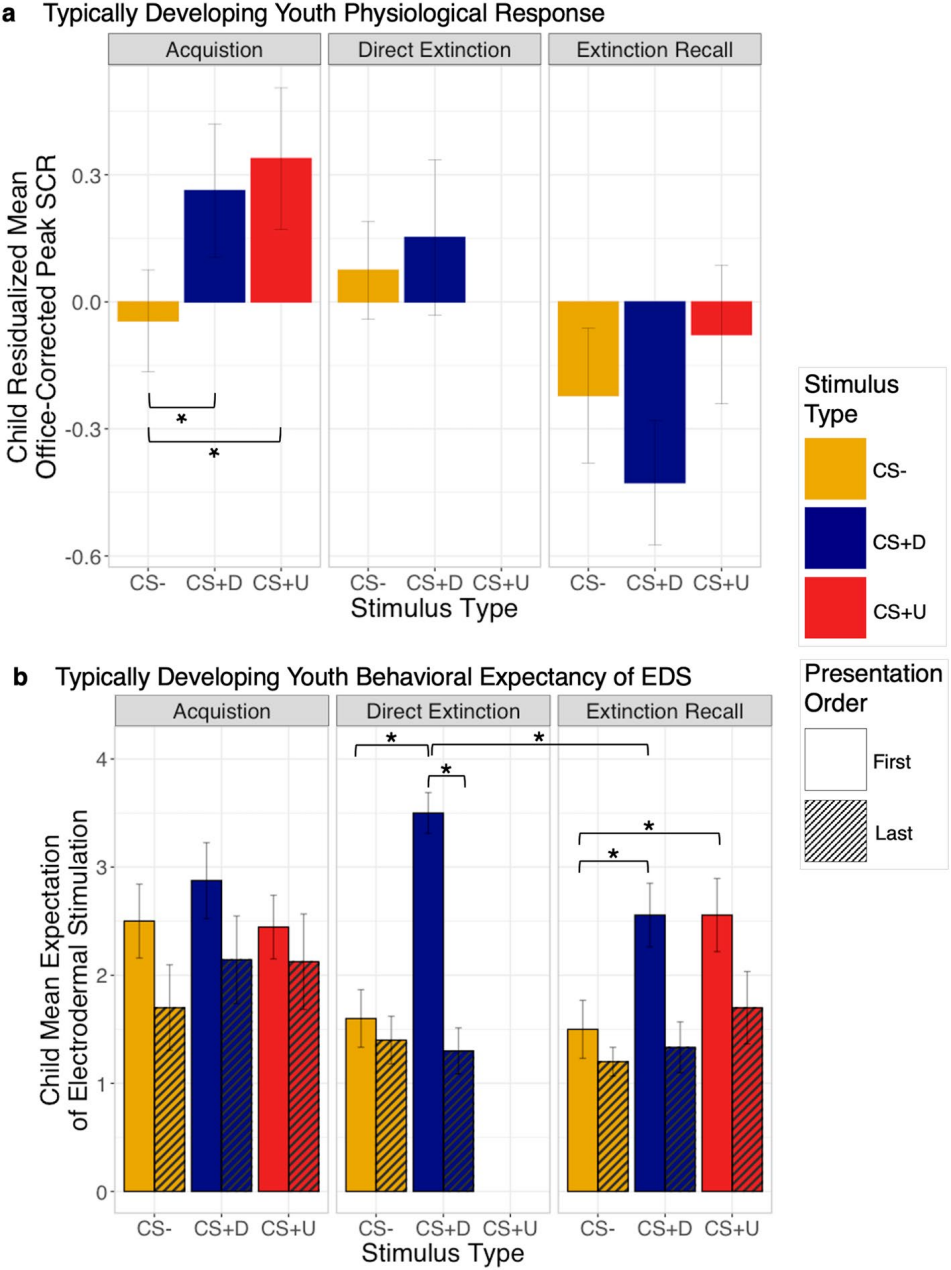
Patterns of SCR and EDS Expectancy during a 3-Day Threat Extinction Paradigm in Typically Developing Youth. (**a**) For each CS type, the mean skin conductance response (SCR) during each phase, residualized for age and biological sex are graphically presented (n = 10). (**b**) For each CS type, the mean EDS expectancy for the first and last presentation during each phase, residualized for age and biological sex are graphically presented. Error bars in all graphs represent standard error. Brackets and asterisks represent statistical significance in Bayesian model 95% HDI. A lack of brackets represents statistical insignificance. *US* unconditioned stimulus, *CS* conditioned stimulus, *CS−* unpaired CS, *CS + D* directly extinguished CS, *CS + U* unextinguished CS, *SCR* skin conductance response

Task validation analyses in the 3-day TD paradigm, visualized in Fig. 3a,b, reveal expected successful acquisition of threat associations and threat extinction. During acquisition on day one, while we detected no significant effects of stimulus in EDS expectancy, we detected increased physiological arousal acquisition to the CS + D (CS + D > CS−, 95% HDI [0.10, 0.48]) and CS + U (CS + U >  CS−, 95% HDI [0.04, 0.45]) as compared to the CS−. During direct extinction on day two, a stimulus by order interaction in EDS expectancy was detected, where youth expressed increased expectancy to the CS + D rather than the CS− during the first stimulus presentation as compared to the last (First CS + D > Last CS + D, 95% HDI [− 3.22, − 1.33]).

During extinction recall on day three, analyses detected no significant differences in SCR response between the CS + D and CS−, suggesting successful threat extinction of the CS + D. Although there were also no detected differences in SCR between the CS + U and CS−, this may suggest an overgeneralization of threat extinction between the extinguished and non-extinguished stimuli. While there was a main effect of stimulus on EDS expectancy, with overall higher expectancy for the CS + D (CS + D >  CS−, 95% HDI [0.36, 2.22]) as compared to the CS−, the magnitude of EDS expectancy was significantly lower during the first presentation of recall versus direct extinction (First Recall CS + D < Last Direct Extinction CS + D, 95% HDI [-1.95, -0.06]), together suggesting reduction in threat response following extinction learning. Further, while we did not detect significant differences between the CS + D and CS + U during extinction recall, youth expressed increased EDS expectancy to the CS + U as compared to the CS− (CS + U >  CS−, 95% HDI [0.32, 2.18]) and similar levels of EDS expectancy to the CS + U during acquisition and recall (Last Acquisition CS + U > /First Recall CS−), where the 95% HDI included zero representing non-significant differences, as would be expected for unextinguished conditioned stimuli. Altogether, explicit EDS expectancy and implicit SCR analyses of the 3-day paradigm were supportive of successful induction and direct extinction of threat responses in adolescents.

### Validation of vicarious extinction learning and PTSD symptomatology

**Figure 4:**
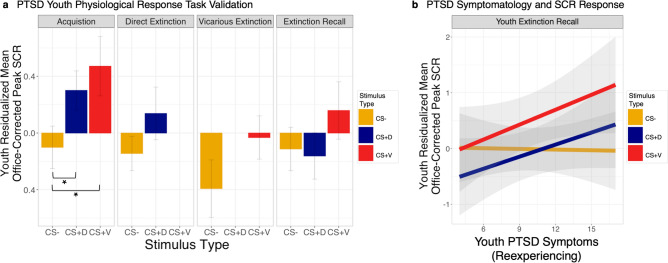
Differential patterns of vicarious extinction learning in youth with PTSD. (**a**) Average youth SCR responses to each CS-type during each phase, residualized for age and biological sex are graphically presented (n = 13). (**b**) A significant main effect of PTSD symptoms and trending symptom by stimulus type interaction is visualized as the relationship between average youth SCR response per stimulus type and PTSD reexperiencing symptom severity (PTSD-RI Subscale B) during extinction recall on day 3, including standard error confidence intervals. SCR responses have been residualized for age and biological sex of the youth participant. Error bars in all graphs represent standard error. Brackets and asterisks represent statistical significance in Bayesian model 95% HDI. * PTSD* posttraumatic stress disorder, *US* unconditioned stimulus, *CS* conditioned stimulus, *CS−* unpaired CS, *CS + D* directly extinguished CS, *CS + V* skin conductance response (SCR).

Within the PTSD cohort now incorporating direct and vicarious extinction, we found similar patterns of successful threat acquisition and direct and vicarious extinction in youth (Fig. 4a). On day one, a stimulus main effect during acquisition in SCR (95% HDI [0.03, 0.35]) and stimulus by order interaction in EDS expectancy (95% HDI [0.64, 2.89]) confirmed CS−US learning. On day two, direct and vicarious extinction were associated with increased EDS expectancy to the CS + D (CS + D >  CS−, 95% HDI [0.18, 1.73]) and CS + V (CS + V >  CS−, 95% HDI [0.30, 2.02]), respectively, and decreased expectancy between the first and last presentation of the CS + V during vicarious extinction (CS + V First > CS + V Last, 95% HDI [− 2.61, − 0.17]).

Finally, no differential SCRs or EDS expectancy between the CS + D/V and CS− were detected during extinction recall on the third day, altogether suggesting successful threat learning and extinction both directly and vicariously for adolescents.

Parents showed largely similar results across EDS expectancy and SCR. Parents express increased EDS expectancy to the CS + D to the last presentation as compared to the first during acquisition (95% HDI [1.00, 3.24]), however no significant effect of SCR. During direct extinction, a stimulus by order interaction on EDS expectancy showed decreasing expectancy between the first and last presentation of the CS + D as compared to the CS + U (95% HDI [− 3.04, − 1.02]), as well as a main effect of stimulus in SCR (95% HDI [0.08, 0.35]) where the CS + D exhibited higher arousal than the CS−. Finally, while we did see a stimulus by order interaction in EDS expectancy during extinction recall driven by both the CS + D (95% HDI [− 1.99, − 0.36]) and CS + U (95% HDI [− 1.96, − 0.33]), the magnitude of EDS expectancy between the first CS + D presentation was significantly higher during direct extinction than extinction recall (95% HDI [− 1.92, − 0.17]), suggesting some successful extinction learning occurred. This is further supported by no significant differences in SCR between the CS + D and CS− during extinction recall, suggesting that the learned threat associations evidenced by EDS expectancy reported above were successfully extinguished by day three.

Next, we examined PTSD-related effects during each phase of this vicarious threat extinction paradigm (Fig. 4b). During youth extinction recall, we detected a significant symptom main effect between PTSD reexperiencing symptom severity and average youth SCR response, regardless of stimulus type (95% HDI [0.001, 0.08]). While a stimulus type by symptom interaction was only nearing significance, this effect is seen in both CS + types, exhibiting positive slopes in youth physiological arousal to both the CS + V and CS + D.

### Parent–child autonomic synchrony during extinction learning

**Figure 5:**
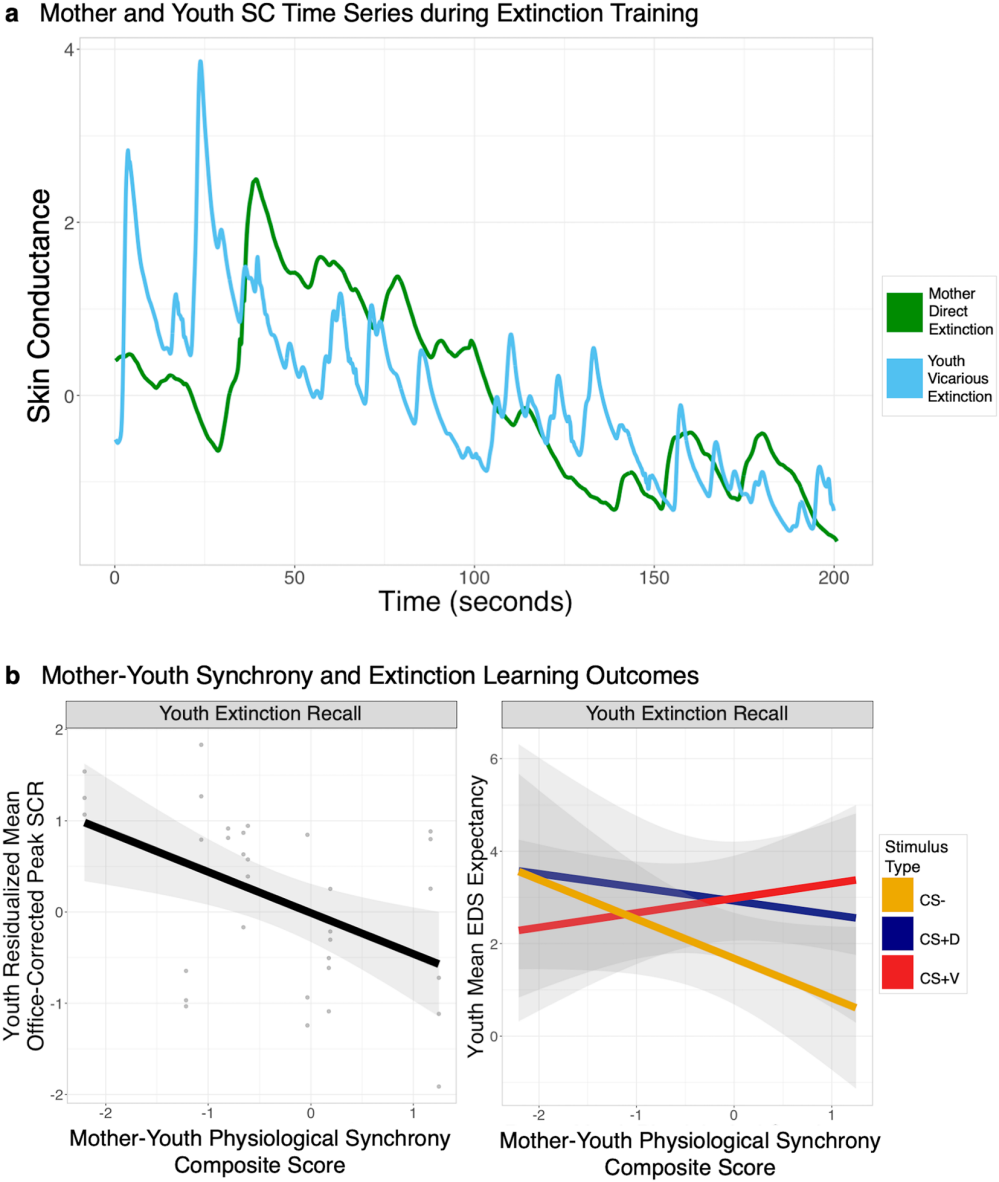
Mother-youth autonomic synchrony during vicarious threat extinction in youth with PTSD. (**a**) A sample time series of skin conductance (SC) during full direct and vicarious extinction training phases in parents (mothers) and youth, respectively. These SC time series serve as the input for autonomic synchrony analyses. (SCR) A scatterplot depicting a significant, negative relationship between parent-youth SCR synchrony during direct and vicarious extinction training and average youth SCR response during extinction recall, regardless of stimulus type. Individual data points for each participant and stimulus type are including with a linear line of best fit. Each variable has been separately residualized for age and for biological sex of youth participants (left). A line graph depicting a significant stimulus by EDS expectancy to the first presentation of extinction recall in parent-youth synchrony (right). Standard error confidence intervals are included (**b**). *PTSD* posttraumatic stress disorder, *US* unconditioned stimulus, *CS* conditioned stimulus, *CS−* unpaired CS, *CS + D* directly extinguished CS, *CS + V* vicariously extinguished CS, *CS + U* unextinguished CS, *SCR* skin conductance response, *EDS* electrodermal stimulation.

Finally, we investigated physiological synchrony in skin conductance levels during extinction training as a possible correlate of PTSD-related differences in vicarious extinction learning, with sample parent-youth time series included in Fig. 5a. When specifically examining vicarious extinction training success during extinction recall on day 3, we detected a main effect of parent–child autonomic synchrony on youth recall SCR (*F*_(11,12)_ = 4.62, *p* = 0.032; Fig. 5b). Parent–child synchrony during vicarious extinction was inversely related to average youth SCR during extinction recall regardless of stimulus type (Fig. 4b). Next, a significant stimulus type by synchrony interaction in EDS expectancy during extinction recall in vicarious extinction parent–child synchrony was detected (*F*_(11,12)_ = 3.62, *p* = 0.02). Here, although the slopes between parent–child synchrony and CS + EDS expectancy (CS + D (t_(11)_ =  − 0.77, *p* = 0.46; CS + V, *t*_(11)_ = 1.28, *p* = 0.23) were not significantly different from zero, this interaction was interestingly driven by the slope between EDS expectancy for the CS−. Synchrony during vicarious extinction learning was inversely related to youth EDS expectancy for the CS− during extinction recall (*t*_(11)_ =  − 2.02, *p* = 0.068). Finally, no such relationships were detected when using a parent–child synchrony derived from youth direct extinction and parent extinction, indicating specificity to vicarious learning effects.

## Discussion

To our knowledge, this is the first study to examine the use of EDS as a US in a classical threat conditioning paradigm, as well as vicarious extinction learning, in typically developing (TD) and PTSD-affected youth. Our findings demonstrate: (1) EDS-conditioning is highly feasible and well-tolerated across TD and trauma-exposed youth, (2) Successful direct extinction learning in TD youth and direct and vicarious extinction in trauma-exposed youth, and (3) PTSD-associated patterns in extinction learning and physiological synchrony between mother–child dyads. Altogether, these findings highlight the feasibility of using EDS and vicarious extinction learning in parent–child dyads and hint at possible PTSD-related abnormalities in direct and vicarious extinction learning that can be expanded upon in future studies. This paradigm also offers high translational potential, mirroring paradigms used both in adult humans as well as animal models to improve our mechanistic understanding of individual and social threat learning in youth trauma-related and other psychopathology.

Our initial cohort of TD youth and mothers confirmed the ability to successfully implement EDS in a classical threat conditioning paradigm in a developmental population with no dropouts. Notably, youth chose similar EDS intensity as their mothers, adequate threat acquisition, and no participant dropout, indicating both high feasibility and tolerability of this classical conditioning paradigm in youth.

A second cohort of TD and PTSD youth completed an expanded 3-day paradigm with and without vicarious threat extinction, respectively. Not only do we continue to report high levels of tolerability of using EDS in a trauma-exposed developmental population, but this is the first study to show feasibility of such a paradigm in youth with PTSD and identify PTSD-associated differences during vicarious extinction learning that may be related to PTSD symptom expression and parent–child physiological synchrony. Altogether, this proof-of-concept study provides strong evidence that a 3-day paradigm using EDS is a feasible and useful tool in future research on the development of threat learning in youths and the expression of pediatric PTSD, that the underlying process of vicarious threat-safety discrimination learning may be mechanistically related to pediatric PTSD through parent–child synchrony, offering potentially novel mechanisms, biomarkers, and therapeutic targets to be explored in future work.

First, we found that an expanded 3-day protocol is overall successful in demonstrating the expected psychophysiological and behavioral correlates of threat learning and extinction in a healthy, developmental sample. Youth exhibited the expected behavioral CS+ differentiation in both threat acquisition and direct extinction learning, as well as lower levels of arousal during extinction recall with no significant differences between the extinguished CS+ and the CS−. Interestingly, youth did not show significant indices of absent or incomplete extinction learning to the CS + U during extinction recall, where we would expect continued increases in arousal to the CS + U due to a lack of extinction training. This may overall suggest that youth have generalized the extinction learning across both CS+ types. This over-generalization effect is consistent with prior research suggesting that when two similar stimuli both undergo US pairing, it is possible to generalize the actively extinguished stimulus to the non-extinguished stimulus^19^.

Within the parent–child dyads of PTSD youth, we then included the recording of parent direct extinction training which was shown to the youth during the vicarious extinction phase. PTSD youth exhibited the same pattern of effective acquisition of threat, with notable PTSD-related relationships in direct and vicarious extinction success and the dyadic process of learning. Youth PTSD symptom severity was specifically positively predictive of arousal to presentation of the CS + D/V during recall, suggesting that both direct and vicarious extinction learning was less successful in youth with higher symptomatology. A youth’s ability to learn safety and threating cues from their parents is especially important for development to learn about the world around them^6,20^. This requires both youth and parents to be able to transmit and receive behavior and emotions effectively. If parents are not able to effectively transmit healthy safety and threat cues, it may lead to behavioral problems or affective disorders like anxiety^21^. One possible mechanism that can help characterize the success of vicarious threat and safety learning is physiological synchrony^22^. Synchrony is the bidirectional coupling of two separate systems, in this case the parent and youth, so that their biological responses are correlated in time^16^ and this time-dependent parent–child synchrony may reflect effective extinction learning. Theoretically, this transmission may be impacted or disrupted by decreased synchrony in youth with PTSD as compared to TD youth. Alternatively, synchrony could be intact between PTSD youth and their parent, and instead may reflect successful learning of heightened parental threat responses during their own extinction learning.

Exploratory analyses of parent–child synchrony in physiological arousal during direct and vicarious extinction, respectively, directly tested these hypotheses. Here, synchrony estimates during vicarious, but not direct, extinction learning were associated with youth extinction recall outcomes. The associations between mother–child physiological synchrony during extinction learning and youth PTSD extinction recall measures implicates potential vicarious learning effects in relationship to PTSD in youth. Notably, higher mother child synchrony was associated with overall lower SCR levels in youth with PTSD during recall, suggesting a general reduction in arousal. This may support more effective threat discrimination (e.g. safety learning), as evidenced by differences in EDS expectancy during youth recall. Here, higher synchrony during extinction learning was associated with reduced EDS expectancy to CS− versus CS + V during youth recall. Altogether this may suggest that social threat learning may be an influential, and potentially modifiable, process in trauma-related disorders for youth, though further study is warranted.

One particularly novel methodology of the current study was the use of electrodermal stimulation as a US in a clinical youth population. This is partly due to the fact that, while threat extinction paradigm designs have been similar across human and animal models, the specific type of US used to elicit learned threat behavior has been less consistent. Electrodermal stimulation has been the most commonly utilized aversive stimulus to elicit learned threat behavior in animal models and adults^23^, while other USs (e.g. air blast to the larynx, loud tones or white noise, or human screams) are more commonly utilized in pediatric studies^24^. While these alternatives may be preferred by researchers who view electrodermal stimulation as “unethical to use with youth because it may invoke distress and discomfort”^25^ these different USs are not necessarily more tolerable. In recent studies in pediatric populations, a “screaming lady” stimulus resulted in 43% dropout^26^, a large noise burst study reported 16.7% dropout^27^, and an air blast resulted in a 14.3% participant drop-out rate^28^. Critically, the current study reports only a 4% dropout rate across cohorts of TD and PTSD youth, with none of the dropouts attributed to tolerability of the US. Therefore, implementing EDS as the US in youth populations may not only increase generalizability and translational potential to animal models and adult humans, but could also prove to be a more tolerable paradigm with decreased dropout as compared to previous similar paradigms.

The underlying neurobiological mechanisms of the vicarious extinction differences exhibited by youth with PTSD are yet to be understood. Previous rodent work proposes the existence of a threat extinction network that involves the amygdala, hippocampus, dorsal anterior cingulate cortex, and ventromedial prefrontal cortex as important nodes in this process^29^. Studies with PTSD have further found that these areas have structural and functional deficits related to the disorder^10,30,31^. Due to this overlap in biomarkers and the preliminary results in our study, including differences in the time-dependent nature of threat learning consolidation in youth as discussed above, future experiments would benefit from inclusion of neuroimaging to explore the possible biological mechanisms that relate to youth, PTSD, and direct versus vicarious threat learning.

While the current proof-of-concept study provides evidence of effectiveness and feasibility of implementation in pediatric and PTSD populations, there were important limitations to address. First, all three of these studies have limited sample sizes. In an effort to mitigate the limitations of small sample sizes, Bayesian modeling was employed to conservatively estimate effects while preserving power and precision. Further, we were unable to collect any genetic information in this pilot. It would be beneficial for future work in this parent–child paradigm to have DNA and RNA to assess the unique role of heritable versus behaviorally modeled/learned threat. On the other hand, the absence of any relationship of direction extinction to PTSD severity, supports the notion of vicarious threat beyond heritable factors. In the PTSD cohort, we collected measures on child psychopathology, but did not collect any information on parent psychopathology symptom severity. This would be useful in future studies to see whether parent psychopathology may be related to or a mediator of youth direct and vicarious extinction success. Future studies could utilize this information in conjunction with genetic information to more comprehensively understand parent–child biological and behavioral interactions. Finally, another limitation is our lack of a trauma-exposed comparison group. While we saw preliminary evidence that PTSD symptoms are related to vicarious extinction learning, we do not have a way to distinguish if this is due to trauma exposure itself or with PTSD diagnosis. Integrating a trauma-exposed comparison group would help clarify any differences in exposure versus more extreme symptoms.

In summary, we report this successful proof-of-concept study supporting a novel paradigm of direct and vicarious threat extinction using EDS as a US within both a normative population of youth, but further within a trauma-exposed population of youth with PTSD. This 3-day paradigm is highly feasible and tolerable, as evidenced by a 4% dropout rate, a rate which is well below other established threat extinction paradigms in adolescent populations. Finally, the paradigm highlights not only successful induction and extinction of threat associations, but it also emphasizes PTSD-related aberrations in the degree and mechanism of direct and vicarious extinction success. We anticipate this novel paradigm to have exciting translational potential across human and animal models that will help to elucidate the mechanisms underlying social learning during childhood that may be disrupted by psychopathology.

## Methods

### Participants

This pilot study recruited a total of 45 youth ages 7–17 years and their mothers to complete a threat conditioning paradigm. The first cohort was recruited to test the feasibility and tolerability of using electrodermal stimulation (EDS) in a typically developing (TD) adolescent population (n = 20) using a 2-day paradigm previously validated in adults. This was followed by a cohort of 25 parent-youth dyads (TD, n = 10; PTSD, n = 15) completing a 3-day version of the threat extinction paradigm to maximize learning and memory consolidation, incorporating vicarious extinction training within the PTSD parent-youth dyads. Typically developing youth were recruited from the community, while youth with trauma-exposure and current PTSD were recruited from local outpatient mental health facilities. Group status (TD or PTSD) was assessed in all parent–child dyads using the Mini-International Neuropsychiatric Interview Screen (MINI)^32^. The MINI-KID has evinced good to excellent concurrent validity on average with other established measures of child psychopathology^33^. Two youth in the PTSD group were subthreshold for current PTSD but were included to maximize generalizability and relationship of study measures to PTSD severity.

Exclusion criteria for all youth participants included past or present substance abuse, brain injury with ongoing symptoms or significant developmental delay, severe/unstable medical condition(s) such as newly diagnosed Type I diabetes or rheumatoid arthritis, acute suicidality, or ongoing exposure to abuse. Exclusion criteria for TD youth included past or current use of psychiatric medication, and past or current mental health diagnosis. Youth in the PTSD group could be currently taking psychotropic medications provided they were not sympatholytic agents. All youth were accompanied by a parent, which was the mother for all participants in this pilot study. All study procedures were approved by the University of Wisconsin-Madison Health Sciences IRB and all activities performed were in accordance with the approved protocol. Parental informed consent and child assent were obtained from every parent–child dyad prior to participation.

#### Procedure

Parents and youth separately underwent a multi-day threat conditioning paradigm, adapted from Milad and colleagues’ (2007) protocol used with healthy and clinical samples of adults^34^. Overall task design is included in Fig. 1 and summarized here. Informed consent was provided to publish the identifiable image in Fig. 1. All participants (parents and youth) completed three phases: acquisition, direct extinction, and extinction recall. During each phase, participants were shown a series of images of a lamp of three separate colors: one represented the CS− that is never associated with a US (yellow), and two CS + stimuli that underwent CS−US pairing during acquisition (red, blue). Threat acquisition phases included lamp images in one context (i.e., on a computer desk), while extinction training and recall were completed with the lamp in a different context (i.e., on a bookshelf).

This study was conducted in multiple phases. The first was an EDS tolerability cohort, where parent–child dyads completed a shorter, 2-day paradigm to test tolerability of electrodermal stimulation as the US in this age group. Here, we only report brief data regarding EDS tolerability and successful acquisition. Next, the primary TD and PTSD cohorts completed a 2-day paradigm. To ensure the 3-day task successfully invoked acquisition and extinction, TD youth and parents completed acquisition, direct extinction, and recall on separate days. PTSD youth and parents completed a similar protocol, only differing by the addition of vicarious extinction training during the second day of the youth paradigm. Vicarious extinction consisted of the youth participants viewing a video of their parent undergoing direct extinction, where they could see their parents’ full upper body and a screen mirror of the stimuli that their parents viewed. The order of extinction training type was counterbalanced across participants. For all parents and TD youth, as only direct extinction training was completed, the second CS−US pairing remained unextinguished (CS + U), while PTSD youth were vicariously extinguished (CS + V) via observation of their parent. Galvanic skin response was recorded throughout each phase of the task.

Tactile electrodermal stimulation (EDS) was used as the US in these paradigms and was delivered to electrodes on the index and middle finger and synched with the experiment in ePrime software (Psychology Software Tools, Pittsburgh, PA). Prior to the experiment, all participants were able to select an EDS intensity between 0.02 and 4.0 mA through a comprehensive intensity calibration procedure. Here, participants had electrodes attached to the index and middle finger of their right hand. They were told to select an intensity that was “annoying, but not painful”^35^, as measured using a 10-point Likert rating scale (ranging from “I feel nothing” to “painful”, with the optimal goal of rating the intensity as an 8/10). The chosen level of stimulation on the first experimental day remained consistent throughout the duration of the study.

### Measures

#### Clinical assessments

For an in-depth assessment of PTSD symptomatology, all PTSD participants completed a series of questionnaires assessing current depression, anxiety, and PTSD symptom severity, including the Mood and Feelings Questionnaire (MFQ)^36^, Screen for Child Anxiety-Related Emotional Disorders (SCARED)^37^, and UCLA PTSD Reaction Index for DSM IV (PTSD-RI)^38^, respectively. The PTSD-RI for DSM-V (PTSD-RI-V) was given to five PTSD subjects due to the timing of the release. For valid direct comparison of the two measures, only congruent questions were used from each version, where subscale and total scores were calculated using only the congruent questions.

#### Outcome variables

For this study, we include three primary variables of interest that encompass both conscious behavioral responses, threat response physiology, and autonomic parent–child synchrony during extinction training: (1) explicit expectation of EDS for each CS type at the beginning and end of each phase, (2) skin conductance response immediately during each stimulus presentation, and (3) within the PTSD group, physiological synchrony between parent and youth during direct and vicarious extinction.

An expectancy questionnaire, adapted from previous threat extinction paradigms^39^, was verbally administered after each phase of the task. Our outcome of interest here was EDS expectancy for the first and last trial of each CS-type: “On a scale from 1 (not at all) to 5 (very much), how much did you expect a shock for the [first, last] trial of [blue, yellow, red]?”. In addition, parts of the questionnaire consisted of attentional checks, where participants were asked whether they received the EDS, which color light(s) they recalled seeing, and which (if any) of those lights were followed by the EDS.

Skin conductance (SC) was collected from the index and middle fingers of the left hand for each participant continuously across all trials during every phase of the paradigm (MP150 recording system, Biopac Systems Inc., Goleta, CA). The skin conductance responses (SCRs) used in subsequent analyses were consistent with previous methods in threat extinction paradigms^18^ by first extracting the peak SC during the 6-s CS presentation for each trial. This value was then normalized by subtracting out the average SC during the 2-s prior to CS presentation while viewing the office scene and a final square-root transformation. For the purposes of this study, only the first four trials of each stimulus for each phase were included due to diminishing response and to be consistent with previous comparable paradigms^18^. Data quality assurance included dropping any participants found to be SCR non-responders, or subjects with greater than 50% of trials with no detected significant above-threshold event-related responses within the stimulus response window (as defined during continuous decomposition analysis) in a particular phase. Using these criteria, no participants were dropped from subsequent analyses.

Finally, parent–child synchrony during extinction training was assessed using full SC timeseries during direct extinction (parents) and vicarious extinction (youth). SC time series data underwent an initial low-pass filter of 1 Hz and 8 Hz down sampling.

### Data analysis

#### Demographic and EDS tolerability analyses

All statistical analyses were completed in R^40^ and RStudio^41^. Demographic differences between TD and PTSD youth in age and EDS intensity were investigated using two-tailed t-tests and Pearson’s correlations evaluated any relationship between age and EDS intensity across all participants. To preliminarily confirm the tolerability of EDS in this mother-youth age cohort, two-tailed t-tests were run to investigate differences in EDS intensity and self-reported stress during acquisition between youth and mother participants. Finally, within youth and parent participants separately, two-tailed t-tests in EDS expectancy between the CS− and CS + in order to evaluate the success of threat acquisition were run.

#### Bayesian mixed effects models

Because small sample sizes in the three-day paradigm may be a hinderance to obtaining meaningful estimates of effects using a frequentist approach (e.g. traditional linear mixed effects modeling), we instead used Bayesian estimation in order to analyze task and group effects to retain power and precision^42^.

As acknowledged by van de School and colleagues, reliable Bayesian estimates are dependent upon information prior distributions. Therefore, models were estimated using the rstanarm package^43^, which emulates traditional model syntax while using Stan (via the rstan package)^44^ for back-end Bayesian estimation. Details regarding this methodology have been previously reported but will be summarized here^45^. Briefly, the 4 steps of this Bayesian analysis include: (1) Model-specific specification of a joint distribution for the outcomes of interest and all unknowns, implemented using the default rstanarm priors, (2) estimate posterior distribution using four chains of Markov Chain Monte Carlo (MCMC), each for 2000 iterations (discarding the first 1000 warm-up iterations of each chain), (3) model evaluation, and (4) visualization of how changes to the predictor affect outcome using the posterior predictive distribution of the outcome given predictors. We report results of steps 1–3 below.

Here, linear mixed effects models (stan_lmer) were used for Bayesian model fitting separately for parents and youth within each phase. For model evaluation, summary statistics of posterior predictive distributions (ppd) were evaluated against the mean skin conductance response for each model to ensure models were successful in reproducing sample means to check for model misspecification, problems with the data, computational issues, etc. Results of ppd evaluation for SCR models can be found in Table 2. Rather than evaluating model outcomes using a p-value^46,47^, the probability of detecting an effect was evaluated by computing the confidence intervals of posterior distributions using a 95% highest density interval (HDI). Here, a credible effect is detected if zero does not fall within the 95% HDI.

**Table 2:** Posterior Predictive Distribution Evaluation.

Group	Age	Phase	SCR Mean	PPD Mean	PPD SE
TD	Youth	Conditioning	0.74	0.7	0.1
		Direct Extinction	0.79	0.8	0.1
		Extinction Recall	0.58	0.6	0.1
PTSD	Youth	Conditioning	0.81	0.8	0
		Direct Extinction	0.75	0.8	0.1
		Vicarious Extinction	0.59	0.6	0.1
		Extinction Recall	0.7	0.7	0
PTSD	Parent	Conditioning	0.47	0.5	0
		Direct Extinction	0.57	0.6	0
		Extinction Recall	0.44	0.4	0

For each SCR model run, the mean and standard error of the mean posterior predictive distribution (PPD) and the mean raw SCR values are reported. All models report plausible mean PPDs/SE, suggesting good model reproducibility of sample data.

*TD* typically developing, *PTSD* posttraumatic stress disorder, *PPD* posterior predictive distribution, *SE* standard error.

Initial task validation models were run to examine the association between behavioral and physiological learning markers and stimulus type. For ES expectancy models, a stimulus type (CS + D, CS + U/V, CS−) by presentation order (first, last) interaction was used, while covarying for age, sex, and subject as a random effect. For SCR, office-corrected peak SCR response was the outcome of interest, while predictors included a stimulus type (CS + D, CS + U/V, CS−), trial number, age, and sex were included as covariates, and subject as a random effect. Sex was not included in parent models as all parents were mothers.

Next, associations between extinction learning and psychopathology were examined in the PTSD group. Depression, anxiety, and total PTSD symptom severity were probed using symptom severity by stimulus type interactions in SCR response. Multiple comparison correction procedures were not implemented due to the exploratory nature of this study. In conjunction with the sample size and preliminary nature of the study, many statisticians have argued that the conservative Bayesian approach may obviate the need for multiple comparison correction across models due to its assumption of a joint a priori distribution on the hypotheses being tested^48,49^.

#### Parent–child autonomic synchrony analyses

Parent–child autonomic synchrony was quantified by recurring properties and patterns of two distinct time series using cross-recurrence quantification analysis (CRQA) via the R package *crqa*^50^ implementing previously validated parameters^22^. Here, parent direct extinction and youth vicarious extinction SCR timeseries were used as primary inputs. CRQA analyses output three highly correlated metrics (Determinism, Entropy, and Laminarity; r^2^ > 0.90). Due to the high correlation between individual synchrony metrics, a Principal Component Analysis was run with varimax rotation to create a single composite score of synchrony using the *psych* package in R to increase interpretability. Using linear mixed-effects modeling, we examined whether synchrony may be an underlying mechanism of behavioral and physiological extinction recall outcomes. Here, we examined whether parent–child synchrony during extinction training (parent direct extinction and youth vicarious extinction) was predictive of youth arousal during extinction recall using average SCR for each CS-type during the first four trials of recall or of expectancy of the EDS during the first presentation of extinction recall. Due to the skew of the recall data, all recall SCR data was log-transformed and then Z-scored prior to modeling. Finally, identical analyses were run using youth skin conductance during direct extinction rather than vicarious extinction to investigate whether synchrony levels are specific to the vicarious paradigm phase or general extinction processes.

## Data Availability

The data that support the findings of this study are available on request from the corresponding author. The data are not publicly available due to privacy or ethical restrictions.
